# Anthropomorphic Capital and Commonwealth Value

**DOI:** 10.3389/fsoc.2020.00024

**Published:** 2020-04-30

**Authors:** Andrea Fumagalli, Cristina Morini

**Affiliations:** ^1^Department of Economics and Management, University of Pavia, Pavia, Italy; ^2^Effimera.org, Milan, Italy

**Keywords:** life-value, network-value, social reproduction, bio-cognitive capitalism, life subsumption, commonwealth, anthropomorphic capital

## Abstract

In this essay, we intend to analyze the process of accumulation of contemporary capitalism, in which the regime of valorization derive from the notion of “common” a “results of social production that are necessary for social interaction and further production, such as knowledges, languages, information affect, and so forth” (Hardt and Negri, 2009) and from its expropriation. When we deal with the concept of “common,” the reference is made to a heterogeneous category. In this text we refer to two modalities of expression of the “common:” the digital common (section network value) and the common of social reproduction (section social reproduction value or the economy of the interiority and anthropomorphic capital). Regarding the first case study, the concept of “network value” is investigated and defined as a product of individual life in a relational context increasingly controlled and subsumed by the social media and big data industry. Regarding the second, we discuss how the activity of social reproduction of individuals is today central in the process of accumulation of the economy. “Social reproduction” is a useful concept to investigate what we call the “anthropomorphic capital,” that is the capacity by the contemporary labor organizations to capture and make productive the essence of today's life and its complexity. In short, it transpires better and better how all activities are productive, i.e., accumulation generators. We observe the apparent paradox of a generalization of surplus value in the era of the decline of waged employment and with it a tension of capital contemporary to the general mortification of living labor. In fact, we note how capital claims to transform the human being into capital itself, explicitly assuming the whole of human existence as a field from which accumulation can be generated (human being, enterprise or human capital). This is what, at this point, we call anthropomorphic capital or the economy of interiority. In the last section, we report some results of an empirical research “Commonfare-Pie News,” able to underline how life is more and more subsumed to the logic of capitalistic valorization, to the point that today we can speak not only of the subsumption of labor to capital but of a real life subsumption.

## Introduction

In an essay that appeared about 10 years ago (Morini and Fumagalli, 2010), we discussed the need to adapt the Marxian labor theory of value to the new forms of accumulation and valorization of contemporary capitalism. After the crisis of the Fordist paradigm, in the last four decades, the processes of accumulation and valorization of the capitalist system have undergone a profound transformation. New factors of production have become ever more relevant in determining the transition to a new type of capitalism, one which we can define as bio-cognitive capitalism (Fumagalli, 2019a). These new factors of production are often the direct expressions of existential processes inherent to human action and its vital faculties, from learning, to language, relationships, feelings and affection. It was in this context that we began to discuss the need to extend and enrich the concept of value by not confining it only to the certified work activity (labor theory of value) but also to the broader spectrum of life (life theory of value). In that essay, the theme of affection and so-called affective work had been the subject of in-depth study, with reference to care work.

In this essay, we intend to continue to broaden this analysis in light of the novelties that have been introduced in recent years in the processes of accumulation of contemporary capitalism, with special regard to “network value” as produced by social media activity and to “social reproduction value,” a broader concept than care or affective work. In paragraph 1, we discuss the concept of commonwealth, a concept that is very often abused, in a particular diction, that is, as an expression of the linguistic and relational practices that are at the base of that social cooperation that is today the main basis of contemporary capitalist valorization and that imposes the putting to labor of life itself. In section network value, we deal respectively with the theme of knowledge and relationships as primary sources of capitalist value (network value), while in sections social reproduction value or the economy of the interiority and anthropomorphic capital we discuss the reproductive commonwealth. Then in the final paragraph we offer some preliminary conclusions by introducing the concept of life subsumption (Fumagalli, 2019b).

## About the Common and Commonwealth: Some Preliminary Considerations

The concept of commonwealth, or the *common* (singular), refers to very different aspects^1^. In the first place, and in general, the *common*, unlike common goods, is not subject to rivalry and therefore to scarcity. This is due to the fact–the second difference–that the *common* is not confinable in a good, but exceeds it, as part of human nature; we can say that, at the limit, the *common* re/produces goods.

As Vercellone, Brancaccio, Giuliani, and Vattimo write:

“The common is not a simple political principle, but a social relationship of production that has its roots and finds its ontological foundation historically determined in the potential autonomy of cognitive labor. This is all the more true if we consider that today one of the fundamental grounds in which capital/labor ratio manifests itself is precisely the development of the productive forces. In short, the social relations of the common innervate the same dynamics of technological and social innovation, and in this very dynamics the common manifests itself as a mode of production” (Vercellone et al., 2017, p. 47).

This quotation aims to underline the fact that in bio-cognitive capitalism^2^ the driving forces of the accumulation are not simply and only based on traditional input like machinery, natural commodities and the labor activity (both manual and intellectual), defined as the capacity of human being to transform physical elements, but more and more on a social relationship, involving the essence of human life itself, regardless of how it is organized.

From this point of view, the *common*, as mode of production, condenses all the characteristics of an input and at the same time is something more, as it is the fruit of a social relationship. It is not a “stock,” rather a “flow.” It creates as an output a commonwealth. For the common to produce value, i.e., to be transformed into a commonwealth, a minimum of organized process is necessary. It is reasonable that, as a social relationship between human beings, the *common* has its own autonomy and its own self-valorization (use-value). The *common* presents itself, in its pure state, as an expression of human vital capacity, but in order to be functional to the process of capitalist accumulation it requires its transformation into exchange value: it requires an organization that can expropriate it.

The *common* is constituted by the vital and cognitive faculties of the human being, from knowledge to the body/soul, from relations to sensations, from language to movement, from sensuality to thought: there is always a production of surplus that derives from the simple fact of existing and living, the moment it is bent to the needs of accumulation. For this reason, the *common* pre-exists cognitive bio-capitalism as much as the surplus-labor pre-exists the system of capitalist production. Bio-cognitive capitalism is able to exploit the *common* only in part and for this reason it needs an adequate proprietary status and to create operative devices to be able to expropriate and capture.

The traditional dichotomy between private and state property needs an overrun For example, knowledge cannot be considered a State asset, since it cannot be expropriated by the individual. But if it remains only the property of the individual and does not socialize, it has no economic and social value.

When Vercellone, Brancaccio, Giuliani, Vattimo define the common as “mode of production,” they refer to Negri's approach (Negri, 2016) and not to Sohn-Rethel's approach (Sohn-Retel, 1977).

Sohn-Rethel speaks of “common production:” that is, the production logic in which “work” and “society” coincide. For Sohn-Rethel “production in common” is the result of a mode of social organization that was present in tribal societies–primitive communism–where there was no private property and the activity of exchange was exclusively aimed at the production of use-value^3^. For Negri, instead, one can speak of the *common* as mode of production when “work” and “life” coincide. And for what to happen, a process of “abstraction” must occur in the labor activity of the two elements that define the power of life: reproduction and language/knowledge. When capital is able to define abstract labor as a marriage of reproduction and language/knowledge, then it is the *common* that becomes the pivot of capitalist accumulation itself and creates wealth (commonwealth). The forms of its expropriation represent the cornerstones of the process of exploitation and valorization of bio-cognitive capitalism.

Starting from this point of view, it can hence be useful to make a distinction between the *common* aimed to directly generate the reproduction activity (reproductive commonwealth) and the linguistic, learning and network activities (cognitive commonwealth). These two types of *common* are strictly interrelated; they are two sides of the same coin. The bridge between them is represented by social relationships and cooperation. The *common* tends to be immaterial, it is an expression of the biopolitical existence of the human being: it's neither rival nor scarce, or better, as such, it is as limited as life and the human race are limited. Knowledge and its diffusion represented the core of the accumulation process in the Nineties during the so-called “net-economy.” In that context, the *common* was able to put life faculties, in particular learning and networking, into the labor performance and, therefore, to transform them in exchange value (cognitive commonwealth).

A further metamorphosis starts to be evident at the beginning of the new millennium with the rise of social media and big data industries. The diffusion of new technologies deriving from and improving AI (Artificial Intelligence), machine learning processes, increasing speed in the classification, and manipulation of data, experiments to artificially create living material (bio-genetics) and so on, represent the way to valorize life directly, without the mediation of labor activity. We can say that cognitive capitalism becomes bio-cognitive capitalism. Bio-cognitive valorization is thus based on two main, among others, factors of valorization: network value and social reproduction value.

## Network Value^4^

The use and collection of data has always been part of human history since its beginning. But it was only with the birth of the industrial revolution that the calculation techniques, refined by the “methodological” break-up by Descartes and Galileo, began to be applied not only to the need to “measure” the physical-natural field (a need that, as is well-known, was at the basis of the development of geometry and mathematics in ancient times, from the Egyptians to the Greeks and Arabs) but also to the control and management of production activities. At the same time, with the advent of the capitalist system of production, we are witnessing the eruption of the “machine” as an immediate productive factor: the act of production (aimed at accumulation) becomes more and more discretionary, detached from the whims of nature, and therefore requires, precisely, one or more units of measurement. The (plus) value produced by the capitalist accumulation needs, in fact, to be known in order to be distributed according to the existing social relationships. As long as capitalist production was mainly material, both in nineteenth century artisan capitalism and in the Taylorist period of the twentieth century, the units of measurement conventionally fixed for the measurement of nature (meter, kilo, liter, volt, watt, horsepower, decimal numbering, etc.) were more than sufficient. When, instead, with the crisis of the Fordist paradigm, production tends to become more and more immaterial and capital more and more intangible, the problem of measurement acquires a dimension that goes beyond the traditional natural geographies. The same sources of valorization are changing and technological innovation, based yesterday on ICT and today on bio-technologies, requires a completely new approach.

Since the spread of information technology, the speed of calculation has exponentially increased. The volume of data created has required, not by chance, new forms of measurement, continuously undergoing redefinition, because they quickly become obsolete. If initially the data-mining techniques were the sophisticated evolution of statistical calculation techniques [and they are studied in this apolitical and neutral perspective, see Giudici (2005); Dulli et al. (2009)^5^], today they are more and more strongly related to personal characteristics, able to define differentiated (individualized) collections of data to be freely traded.

A well-known example, on which Matteo Pasquinelli has dwelt, concerns the Google Pagerank algorithm (Pasquinelli, 2009a). This is how this algorithm is described by Carr (2008):

“At the heart of [Google] is the PageRank algorithm that Brin and Page wrote while they were graduates student at Stanford University in the 1990. They saw that every time a person with a Web site links to another site, he is expressing a judgment. He is declaring that he considers the other site important. They further realized that while every link on the Web contains a little bit of human intelligence, all the links combined contain a great deal of intelligence far more, in fact, that any individual mind could possibly possess. Google's search engine mines that intelligence, link by link, and uses it to determine the importance of all the pages on the Web. The greater the number of links that lead to a site, the greater its value. As Jonh Markoff put it, Google's software systematically exploits human knowledge and decisions about what is significant. Every time we write a link, or even click on one, we're feeding our intelligence on Google's system. We are making the machine a little smarter and Brin, Page and all of Google's shareholders a little richer” (p. 27).

The algorithm, today, is establishing itself as the expression of the *general intellect*, as its phenomenological expression. An expression that varies and is flexible according to the field.It does not directly concern the bios but the cognitive (Fumagalli, 2017). Today it is the instrument for measuring the value of cognitive intensity. It is, at the same time, a real and formal subsumption. But it is also something more. It is a mathematical measure of network value, able to condense wetware and netware on the basis of software. It is therefore the basis of accumulation and enhancement.

“What PageRank identifies and measures is a network value in a very numerical form. If a commodity is traditionally described by a value of use and an exchange value, the network value can be considered an additional level attached to the previous ones that describes the network of social relations. This term can be somewhat ambiguous as it can be misunderstood as the “value of networks” according to the much celebrated “wealth of networks” described by Benkler (2006). On the contrary, a notion of network added value should be introduced here for the sake of clarity^6^. In fact, PageRank produces what Deleuze and Guattari (1972) described as machine surplus value by referring to the surplus value accumulated through the cybernetic domain, i.e., the transformation of a code surplus value into a flow surplus value. Through PageRank, Google has not simply gained a dominant position in the control and possession of extensive web indices, but above all a monopoly in the production of such network value” (Pasquinelli, 2009a, p. 9).

The example cited is paradigmatic of the evolution of contemporary valorization processes that, starting from the cognitive, have increasingly pervaded the *bios*, to the point that the evolution between human being and machine tends to increasingly diversify along two parallel and synergic directions: the relationship between subjectivity and machine and that between physical body and machine. Much has been written about the former, starting in the early 1970's when the relationship between mind and machine was investigated. And it is on this hybridization that Franco Berardi coined in the early 2000's the term *cognitariat* (Berardi, 2002, 2004). The definition provided by the Garzanti dictionary (“precarity of those who do intellectual labor”^7^ does not capture the whole complexity of the term. It is in fact the concept of intellectual labor that is put into question. If in the last decade of the last century, we can see a sort of “Taylorisation of intellectual labor and intellectualization of manual labor” (Fumagalli, 2017), today this process has gone far beyond the dichotomy, albeit redefined, between manual and intellectual activity, and has overcome this difference. A difference that has been included within the term “cognitive labor” and expanded into that of “relational labor.”

In fact, it is from this labor that the value of the network originates, which today tends to pervade, in differentiated and diversified ways, different productive activities, from logistics (increasingly digitalized), to shopping centers and up to immaterial consulting. Everywhere there is an app, there is network value, that is, biopolitical value.

In the face of recent developments, Romano Alquati's observation of the value of information at the time of the Olivettian factory is extremely topical, with reference to the Taylorist context:

“The productive work is defined in the quality of the information elaborated by the worker to the means of production, with the mediation of the constant capital” (Alquati, 1963, p. 121).

The value of the network is at the same time the result of a process of exploitation (Fumagalli, 2017, 2019b), extraction (Mezzadra and Nielsen, 2017), and imprinting (Chicchi et al., 2016). It is the form of surplus-value of the cognitive, to which it will be necessary to add the surplus-value of the bios. It is the result of the interpenetration of the human sensory system with the informational and digital network that increasingly envelops the activities of production and accumulation. From this point of view, we are witnessing the machine becoming more human-like (Braidotti, 2013), the spatial (or rather, relational) becoming more human-like (Pasquinelli, 2009b)^8^, but at the same time, the human becoming more machine-like (Raunig, 2010, 2016; Fumagalli, 2017).

The creation of network value, through the processing of Big Data, takes place mainly in some sectors. The data, in itself and for itself, is characterized by use value, such as the labor power or the common (in singular, Fumagalli, 2017; Vercellone et al., 2017). As productive input in an immaterial production context, it is transformed into an exchange value, within production contexts able to use the appropriate algorithmic technology. Such a process, however, is far from being homogeneous and precise. In fact, in the management of the clouds of Big Data, confusion, approximation, and heterogeneity reign, as does the imperfection of the technologies, continuously in process, thanks to the involvement of the same suppliers and users of data. Consider, for example, the translation service offered by Google: the difference with other systems is precisely the use of a larger and very chaotic dataset. It does not provide a word-for-word translation, but an analysis of millions of official texts that come from sources such as the UN and that provide a large amount of data:

“Despite the confusion of input, Google's system works better. Its translations are more accurate than those offered by other systems. And it is much, much richer. By mid-2012 its dataset covered over 60 languages. It was even able to accept voice input in 14 languages to make translations smoother. And because it treats language simply as a chaotic mass of data to which to apply probability calculation, it can even translate between two languages like Hindi and Catalan” (Mayer-Schonberger and Cukier, 2013, p. 76).

Exactness plays a secondary role, after vastness, in identifying the general trend and capturing the whole phenomenon. Take for example the disordered and flexible mechanism of “tagging,” widely used on Internet. This system allows users to label mainly photos or videos, making it possible to trace different content on the network through tags created by users. The confusion in this case may be due to the wrong writing of the tags and the way they are organized. Therefore, the managing and the governance of such large amounts of data need a specific business section that it is called Business Intelligence (Dataskills., 2017). It is a business function that has the function of extracting value for the different productive purposes from the processing and distribution of data (Camiciotti and Racca, 2015).

It refers: “to the set of business processes to collect data and analyze strategic information, to the technology used to implement these processes and to the information obtained as a result of these processes” (Camiciotti and Racca, 2015).

Business Intelligence is therefore a system of models, methods, processes, people and tools that make possible the regular and organized collection of data generated by a company and through processing, analysis and aggregation transform data into information that is storable, retrievable, and presentable in a simple, flexible, and effective way to support strategic decisions, tactics and operations.

**Figure 1:**
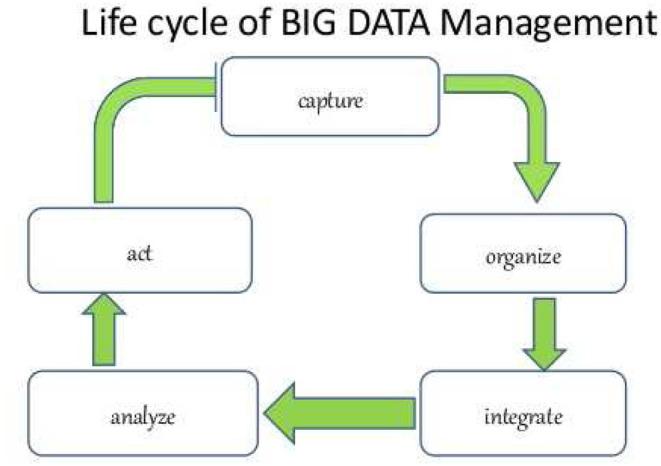
Reproduced with permission from the author: Takrim Ul Islam Laskar, www.slideshare.net.

The Business Intelligence system therefore involves (see Figure 1):

- the collection of the data of the company's patrimony
- their cleaning, validation and integration
- subsequent processing, aggregation and analysis
- the fundamental use of this amount of information in strategic and valorisation processes (Dataskills., 2017).

The structure of the real life cycle and valorization of big data systems can be described in the following figure, on the basis of a succession of operations that begin with the “capture/appropriation” of data, their “organization,” “integration,” “analysis,” and their transformation into “action.”

In most cases, especially with respect to unstructured data (about 80% of the total), these data are presented as use value, produced and socialized by users/consumers in the performance of the acts of cooperation and relationship that are carried out daily. It is not by chance that we speak of capture, or rather of moderately forced or voluntary expropriation.

This life cycle describes, in a nutshell, the process of valorisation of big data. It is worth considering the two operations of “organizing” and “integrating.” These are two operations that only in recent years have been able to reach a certain degree of sophistication, thanks to the technological evolution of the 2nd generation algorithms. The organization and integration of the data is at the base of the production of the network value. It is the productive aspect of exchange value, while the “analysis” and the “action” represent its commercialization, that is the monetary realization on the outlet markets.

It is in these two phases that “platform capitalism” begins to structure itself. With the term platform capitalism, we intend an organization of labor and production in which the demand-supply ratio is intermediated by an algorithm and a digital platform (Srnicek, 2017). In this new context, companies need to define a new capital composition able to manage an increasingly automated process of data division according to its potential commercial use. It is based on the more or less conscious participation of individual users, now transformed into *prosumers*. It is in fact the users of the different platforms, whether they provide information to satisfy desires or virtual spaces for communication, play and development of relationships, that provide the raw material that is then subsumed in the capitalist productive organization.

We can say that if today human relations, social cooperation, the production of collective intelligence, and social reproduction are the expression of the common as a mode of production (Negri, 2016; Fumagalli, 2017; Vercellone et al., 2017), at the present time they are the basis of the communism of capital, that is, the capacity of capital to subsume and capture the instances of life of human beings^9^. The main tool of this ability to capture the common human being is *machine learning*. Until recently, very few people knew what algorithms were, while today they are present in everyday life as a fundamental aspect of modern society:

“They are not only in your mobile phone or laptop, but also in cars, at home, in your appliances and in toys. Your bank is a gigantic web of algorithms and humans just turn a few knobs here and there. The algorithms decide the time of the flights and govern the airplanes. They operate the factories, buy and ship goods, collect the proceeds and keep the accounts. If all of a sudden all the algorithms stopped working, it would be the end of the world as we know it” (Domingos, 2015, p. 32).

The algorithms work without us realizing their presence and functioning. By algorithm we mean “a calculation procedure” or a method for solving a problem, “a sequence of instructions that tells a computer what to do” (Domingos, 2015). Algorithms are the basis of machine learning. To understand the power of machine learning we can use an analogy reported by Pedro Domingos, in which this type of technology is compared to agriculture: the learning algorithms are the seeds, the data are the soil and the programs learned are adult plants. The machine learning expert is the farmer who plants the seeds, irrigates and fertilizes the soil without interfering further. Thanks to this metaphor, two aspects emerge. The first is related to the large amount of data, because the more we have, the more we can learn about it. The spread of machine learning is closely linked to the appearance of Big Data. The second aspect, on the other hand, shows how a large amount of available data can increase the velocity that characterizes these processes. With machine learning, the process undergoes a strong acceleration:

“The Industrial Revolution has automated manual work, and the Information Revolution has done the same with intellectual work. Machine learning, on the other hand, automates automation itself: if it were not there, programmers would become the bottlenecks that hold back progress” (Domingos, 2015, p. 12).

Algorithms help us navigate through the vast amount of data on the net, but above all they can influence our decisions and the cultural context. The ones that do this most are the algorithms of social networking platforms: every time we use them, we leave information that is recorded, processed and used for other users. The collection of individual information is then sent to the community. As the algorithms suggest what we like and help us in our relationships, they begin to shape our identity and influence our choices (Fumagalli et al., 2018). In the information society, the main problem lies in more or better, in the unlimited choice that Big Data creates: among the multiplicity of products to choose from or occasions to seize, which one can be the most suitable for us? Algorithms and machine learning offer a solution. In the same companies, the number of operations to be carried out increase exponentially over time, as do the number of customers. As a result, machine learning becomes fundamental:

“Amazon cannot properly encode the tastes of all its customers in a program, and Facebook is not able to write a program that chooses the best updates to show to each of its users. Walmart, the giant of distribution, sells millions of products and has to make billions of decisions a day: if its programmers tried to write a dedicated program, they would never end. The solution adopted by such companies, on the other hand, is to unleash the learning algorithms on the mountains of data they have accumulated and let them guess what the customers want” (Domingos, 2015, p. 52).

These algorithms are not perfect, but in providing their results they affect the user and his decisions. They are the intermediaries between the data and the consumer and concentrate power and control within them. They are the modern-day assembly lines.

## Social Reproduction Value or the Economy of the Interiority and Anthropomorphic Capital^10^

Social reproduction plays an increasing and paradigmatic role in bio-cognitive capitalism. It represents the main factor of the enlargement of the accumulation basis.

The contemporary (re)production context is mainly based on processes of exploitation and control of the organic (Cooper and Waldby, 2014; Villani, 2018) and the emotional (Hochschild, 1983) aspects of bodies-mind. It refers to a capitalist paradigm based on forms of social reproduction, or directly of social production, observing the meticulous tendency of capital to deepen the mechanisms of extraction of surplus value through an expansion of the fields to which it applies its domination. It happens due to multiple processes of abstraction and mortification which affects a multitude of concrete and living activities (originated by needs, therefore marked by the use value, not immediately transformed to exchange value); they are passivated by capital in order to reproduce itself, that is, becoming capable of directly producing accumulation. We observe that more jobs, linked to needs, affections (*affĕctu(m)*, derived from *afficĕre* “hitting, provoking a state of mind”), knowledge of bodies-mind, today explicitly produce value for capital, while remaining unchanged the fact that these services remain, as yesterday, placed outside of wage mediation.

The concept of labor has been described by Marx as an expression of capacity, of power in the worker's corporeal existence, and of production as a process of intentional transformation of nature in order to produce the tools of his own existence^11^; “the worker is in relationship with his labor [] as a foreign object” (Marx, 1990, p. 227) but also in the sense that labor “cannot exist without the objects on which to practice. In a certain way, it is clear and precise, in this context, the identification of materiality, appropriability, exchange allowed by the material labor of the worker. However, Marx anticipates the overall mortifying, alienating tendency of capital, since in this objectification, in this production of external existence that is fixed in the object into which the worker already puts “his inner world,” “his life, which no longer belongs to him but to the object” (Marx, 1990, p. 227). And then it will come to appear that “a growing number of functions of the labor power is grouped in the immediate concept of productive labor, and an increasing number of people who perform it in the concept of productive workers, directly exploited by capital and subject to its process of production and valorization” (Marx, 1969, p. 749).

Thus, the reflections of Marx on alienation in the *1844 Manuscripts* are at the root of the problems with which we are confronted in the contemporary world. In times of exploitation of an ever-increasing mass of workers placed outside the wage relationship by the generalization of labor precarity and by technological innovations, the reflections of Marxist feminism, insisting on the concealment of women's work in reproductive pathways (Dalla, 1972) and on the decisive role played by reproduction for accumulation (Federici, 2004), remain fundamental and continue to offer inspiration. Alisa del Re in an issue of *Viewpoint Magazine* dedicated to Social Reproduction helps us to define the broad field we are talking about and the spheres in which it is applied:

“The reproduction of individuals can have different connotations: biological, material, emotional, cultural, relational. It is obvious that all these aspects are generated by an historical social context and at the same time they characterize it” (Del Re, 2015, p. 4).

The great novelty of the current paradigm of social production–in this phase of history and society–lies in its capacity to extract economic value exactly from these different connotations of the human capacity to re-produce itself (biological, material, affective, cultural, relational), that is, exploiting precisely the capacity to “take care of” or even “pay attention to.” It is to be understood as a broad action of relating and communicating the subjectivity allowed by language, guaranteed by the new machines based on artificial intelligence that have made possible a totalization of the labor capacity (Berardi, 2016). What stands out is the increased alienating force of capital, which, by placing reproductive matter at the center of processes, risks to generate forms of human self-alienation.

The various digital devices act as stimulators and catalysts for the social production process. The social factory has, in a certain sense, been concentrated in a smart-phone, which condenses messages of love and data of all kinds, eradicating attention and paid services with free apps, perennial availability and personalized induction to consumption, definitive control of movements through GPS, quantitative evaluations of the body (steps, beats, hours of sleep). It is a factory that we buy and maintain ourselves voluntarily, that allows us to have news in real time and to keep us connected to the rest of the world, to which we give (all) our time (life). An offshoot of bodies that de-realizes bodies and dematerializes their actions. Deprivation of social knowledge allowed by algorithmic governance (Baranzoni and Vignola, 2016). Inter-passiveness induced by the dependence on the stimulus and by the communicative excitement (Fisher, 2009), which moves affections, that is, generates states of mind.

To better examine the complexities of the present it can be useful, above all, to find suitable suggestions to understand the general enlargement of the regime of gratuitousness of the current re-productive work performance, so full of subjectivity and social connections as it is. Since the substance of labor today also resides (and not only, obviously) within ourselves, it is part of the bodies-mind of human beings; we try to make the economic value coincide with the value of human beings itself, it is therefore the *life value* (Morini and Fumagalli, 2010). Life that is worth if objectified, recognized, made visible, taken as a model by others, followed by followers, confirmed by the metrics, by the number of quotations. The capital earns thanks to the photo of and information on your private life (births, marriages, holidays, deaths,…): you have thus created an *economic ego* (Cesarano, 1979, p. 7).

Moreover, since, returning to Alisa del Re, the matter of reproduction is “biological, material, affective, cultural, relational,” we are confronted, also in this case, with the rigidity of reproduction: one cannot leave, refuse, if not leaving, rejecting, parts of oneself or of the worlds, of the forms of life to which we are linked, which recognize us, with the risk of remaining isolated. Today it is the social person who is the collector of the value produced in the contemporary world, with all its organic sexual corporeity, including linguistic, that is, emotional and relational abilities. It is an extraordinary complication. Alienation from the object yesterday created a relationship with a foreign object, enemy, independent of him (outside); today this object of production is (can be) part of the Self, inside the communicative carousel of the new machines, bringing alienation into the worker, the worker herself (inside). Will this be the heart of the psychic malaise that seems to pervade the greedy Western society? What creative, imaginative effort do we need? How do we get out of this inter-passiveness? How to find networks instead of platforms, real communities instead of virtual communities?

It is also essential to take into account the growth of an economy of new reproductive services to the social person, aimed to reduce life time and make it more productive: it is a clear effect of the regime of social production of social work: the majority of platforms (from Arbnb to Deliveroo, from Uber to Amazon) are based primarily on the provision of reproductive services and leisure (ready food; houses, cars or bicycles for rent; tourism; online shopping…). These platforms are part of the framework of the libidinal economy or of the interiority that constitutes the real engine of contemporary accumulation.

As the productivity of the industries which take part in the reproduction of the labor power increases, we see how the establishment of the gratuitousness of the living work of reproduction represents today a determining factor in lowering the value of the whole labor power, and therefore in the increase of the surplus value. The tendency to generalize the gratuitousness of work is not only the effect of the generalization of precariousness. It is the most precise indicator of the contemporary confusion of levels brought about by the economy of interiority, which takes us “beyond the formal dominion of capital” within its “real dominion” (capitalist realism), where there is no separation between structure and superstructure, “circulation of ideas and circulation of goods, being both causes and reciprocal effects in a concatenation that repeats the serial module of the bolt and the vine” (Cesarano, 1979, p. 8).

If, as Christian Marazzi has pointed out^12^, in the emerging anthropogenetic model of contemporary capitalism, the “living being” contains within itself both the functions of fixed and variable capital, “that is, of material and instruments of past work and of present living work.” We can talk about an anthropomorphosis of capital. We can glimpse the risks of a paradoxical alienation of the subject from his own existence to live the life that capital imposes to live, in a passive way. There is therefore a danger: the activity to which the system would like to oblige everyone within the new mesh of the present economic paradigm, risks to abstract people from themselves, forcing them to adapt to a know-how, which is functional to the society of performance. In this false movement, affections (i.e., to “do something for”) are integrated in the current macroeconomic process, within a sort of “interiority economy.” The first effective representation of this process was, as mentioned, the domestic labor of women, where the value was taken from the work of the mother or wife and taken out of consideration of the salary for the male worker. We owe to feminism the understanding of what has happened and what is happening even more intimately today, that is to say the risk of an integration of life, with its scope of relationships, sexuality, knowledge, education, care within the cycle of capitalist production. Every aspect of social life risks being selected by the logic of capitalist valorization, in the same way indicated by the model of reproductive work, feminized, historically incarnated in the bodies of women.

## Commonfare-Pie News Project: Some Qualitative Results on the Perception Of Precarious Condition and Emerging Needs^13^

In this paragraph, we illustrate some first empirical findings, the analysis of which forms one of the sources of the theoretical framework we illustrated in the previous sections. The dataset is provided by the European Horizon2020 research project “Commonfare-Pienews”^14^. The aim of the project is to create a collaborative platform capable of networking some good practices of self-organization of Welfare from below in the three pilot countries considered (Croatia, Italy, Netherlands). In order to achieve this objective, field research was conducted (Pie News Report., 2017), to determine the emerging needs of some segments of the population deriving from precarious labor conditions^15^.

First of all, the 252 interviews show the dominance of the perception of a job as segmented, devalued, and humiliating. Even in the Netherlands, where there is a more advanced welfare system than in the Mediterranean, the interviews highlight the progressive disappearance of work (“jobs are simply disappearing”), and the desire to be able to reject some trivial jobs, investing their time instead “in projects that I really believe in” (Pie News Report., 2017, p. 71).

In the interviews, this type of capture process partly worked for a first group of precarious workers who invested a lot of energy in the work environment, absorbing a lot of rhetoric related to the participation and creativity of new jobs. In the Italian context, it has been estimated that this first generation of precarious workers, defined as postfordist or first generation, includes a group of people aged between 30 and 49 years. They experienced the beginning of the transformations of a job that, on the one hand, went beyond the cornerstones of the Fordist enterprise and its iron organizational disciplines, meeting the desire for autonomy of the subjects, and on the other hand it was loaded with new investments, passing from the ethics of “obligation” to that of “self-realization” (*travail-self-fulfillment*) (Meda, 2016, p. 11). Between technological innovations and higher professionalization, work gradually becomes the field where one's skills can be perfected (Meda, 2016, p. 12). On this subject, in the PieNews report relative to the Italian case (Basic Income Network Italia., 2018, p. 55), we speak, in fact, for this sample, of “construction of the imaginary” and “voluntarist efforts;” of “sacrificial overload” and of “continuous performanceism” (Basic Income Network Italia., 2018, p. 55).

However, looking at the results of the interviews and focus groups carried out in the Italian context, it should not be forgotten that the first generation of precarious workers is still politically aware. The language of rights, protections, guarantees of welfare, of some practices of struggle and social claim, on which the political discourse of the traditional workers' movement was based for decades, is still alive and present (Pie News Report., 2017, pp. 48–49). By this we mean to refer to networks of proximity and political and trade-union affiliations, to experiences of militancy and activism which strengthen knowledge and trust, to the capacity of the subject to position himself with respect to the socio-political context, in spite of the imperative of individualization introduced by precariousness. In fact, we will also talk about a precarious point of view, referring to the need to create knowledge useful to make a correct diagnosis of the situation: experience becomes a method of discourse that can never be completely constrained by power relations (Harding, 1986).

### The Precarious Inclusion

This condition is explicitly described as a battle, or even a war, by Edoardo, a 41 years old man who works in the arts sector:

“In the 1990's, in order to cope with extreme job insecurity and intermittent employment we were facing; we were very aggressive and savage. It was as if we were ascetic-predators. We were nomads, we constantly moved where we could plunder anything, always carrying the burden of anxiety […]. We followed whatever could bring in income, wherever there was funding you could find us. We put on a sort of tortoise shell to protect ourselves and to be able to deal with the labor market. We found a way to survive but to the detriment of social and emotional dimension.”

These precarious workers were, therefore, consciously in search of a pro-active approach to flexibility which could improve their working and living conditions, and thus they carried out many activities and nurtured many interests in order to achieve greater autonomy and independence. However, between the construction of the imaginary and voluntary efforts, an element of “compulsion” simultaneously emerged, namely the sacrifice required to distinguish themselves in the jungle of job insecurity. As Costanza highlighted:

“In order to face critical situations in my life I had to roll up my sleeves and fight, even though it means being totally dependent on work…I had been working harder…I will have to work forever…I had to work also to help my parents (my mother is a widow now…), I gave up the idea of having a child because of my job. Working becomes a full exploitation […] I am totally servant of my master, of the firm I work for […] work has totally bought me.”

Matilde, 38 years old, talked about the obligation for “continuous job performance,” and Mattia, 45 years old, pointed out how this continuous solicitation resulted in psychological fatigue, hence the need to introduce forms of psychological counseling:

“They should invest in social services in the coming years. After having worked for 14 years in the television industry, I have absorbed so much discomfort associated with work that I think psychological counseling is necessary.”

When Monica (45 years old, teacher) said “those who cannot stand the loop are doomed to drop out,” she perfectly described as work has been, willingly or by force, the center of gravity of precarious lives, in the effort to keep the pace requested. This tension resulted in a pervasive strategic individualism, as Alessandro said:

“The transient experience we live is certainly not a condition of well-being. It rather forces us to be always otherwise intelligent. For sure, today's work is mainly based on challenge and conflict with others: I succeed only if I lose myself in my work, thus resulting in isolation and absence of relations.”

From the early years of the new Millennium, when talking about precarious workers one can speak of “second-generation” or “native precarious workers” as well as “crisis-related precarious workers” (Gobetti and Santini, 2009, 2016). It is the youngest generation: people aged between 18 and 34. They were born and raised in the time of job insecurity and crisis, and they are fully engaged in “occasional odd jobs” first and in the gig economy later. They seem to have a more disenchanted, less “ideological” and more pragmatic approach to work. There is no longer inside and outside, there are no *standards* one wishes to stick to in order to exit the precariousness which is the denial of a reference model: in fact, the precarious worker, from the semantic point of view, encompasses first of all a lack of identification; he “belongs to the sphere of “non,” he is exposed to anything resulting from precariousness, he is on the edge of risk” (7Blu., 2005). As precariousness is institutionalized, it becomes the norm, the atypical turns into typical, it is maybe easier to assess some “tricks” more immediately: precarious workers are less emotionally invested in their work; they are aware that their job will not help them to fulfill themselves or gain social mobility; they face the challenge and risk of job insecurity in a more positive and above all concrete way.

### Life Itself Is Put to Work

This generation seems to be doomed to this condition which has now become structural andhas permeated life as a whole to such an extent that “work has invaded all aspects of social life, it ended up swallowing space and time and conquering *the whole life*” (Aronowitz, 2006, p. 58).

“If I had to express my working time as a percentage of my financial condition I should say that 99% corresponds to the former and 1% to the latter….besides, when I finish working for the day I keep thinking about work” Luca, a freelance architect, told us, and Giorgia said, echoing his words: “*It is very difficult to quantify my working hours. I think this happens to everyone…I feel like I never stop working.”* Despite the effects of this pervasive dimension, a first element of novelty emerges from the words of Alice who introduces an issue that we often found in our focus group meetings:

“I do not want to do without my life, my relationships and my activities beyond work, which become discriminating factors to relate to work itself.”

There is no reference to the previous labor guarantee systems, as Stefano said:

“We are no longer in the phase where the workplace allows us to build social relationships that lead us to recognize each other.”

Fordism and the rights it entailed seem to be definitively gone, historicised.

Native precarious workers face the problem of an economic crisis which is no longer linked to society and its actual needs, and therefore it does not know what to produce and why. Such an economy is just as uncertainty about its capital accumulation process. The content of work seems considerably devalued and standardized.

### Beyond the Ideology of Work

However, the feeling of permanent and universal randomness in daily life has resulted in a change in terms of planning, thus contributing to inflict a heavy blow on the ideology of work. Native and crisis-related precarious workers are increasingly engaged in occasional and contingent work. As a result, the bundle of affection, social, relational and communication skills that the Post-Fordist precarious worker was still willing to put in the production process, seems now largely transferred beyond and outside of work, thus reconfiguring work attractiveness and regarding work as a mere activity that ensures the reproduction of the material conditions of existence. This situation is described by Alice who said:

“People who work in the same workplace do not know and recognize each other. In the last three years, in my workplace there has been a high turnover of staff to such an extent that the changing room lockers are completely scribbled because of the many names written and erased on them…this high turnover of employees does not allow to start a relationship.”

Native and crisis-related precarious workers are contemporaneous with the descending parabola of the ideology of work, as work is no longer regarded as the only factor of self-recognition and subjectification. Precariousness is perceived as an inevitable horizon and, consequently, the effort to be made is to bend it to one's individual needs.

Without ideologies and with pragmatism, the new precarious subjects frankly wonder whether, in the current crisis, it is convenient or not to activate themselves into work^16^.

In this regard, Francesca, a student engaged in odd jobs, said:

“I know people who have accepted bad job offers, without any rights and with absurd working hours. I do not want that for me and above all I do not want to do the same job all my life as well as I would not like to do a job that does not interest me. I wish I could turn down a job offer that allows me just to survive.”

The mass casualisation of employment involves the end of the centrality of work; home, income, time, recognition of civil and social rights, these are some of the common needs expressed that may trigger a new political discourse.

### Impermanence (Morini, 2015) and Lightness of Native Precarious Workers

This continuous transition experienced by precarious workers seems to require a greater *lightness* despite the highest levels of intermittent work and poverty in terms of income (most young people aged between 18 and 24 years earn between 1,000 and 5,000 Euros per year). Caterina, 29 years old, a University teacher who is employed on a precarious contract, said to hang in the balance between “resignation and a sort of gratitude for having a job even if it is temporary;” Cosimo, 24 years old, added: “I do not regret not having a permanent job. Working all day is not my aspiration.”

Native precarious workers do not define themselves in terms of lack (of income and employment for instance) or absence (of answers or certainties). These elements have characterized the stories of the first-generation precarious workers who tried to manage a paradigm shift and who were pioneers of that sort of anxiety which resulted from the uncertainty of the present and the future. Native precarious workers were born in a post-wage-based society, and in addition in “the time of crisis” (international, economic, financial, etc.): they have not experienced conflicts between abundance and scarcity, they have taken their first steps on fragile terrains and on such terrains they have learnt to walk with *lightness*^17^. This situation becomes even more evident in the complicated dialectics between generations as Anna highlighted:

“for my parents it is unthinkable that at the age of 25 I am not able to see the next steps that are aimed at building paths for my future. Although I have a job–I am self-employed–I do not see my future so different from my present.”

Learning to walk with *lightness* puts us in front of young precarious workers who are very active, who are able to manage their time and relationships and adapt them to the contexts they live. Then again, although in Italy second-generation precarious workers suffer the highest levels of unemployment, it seems they better understand the risks of a design that induces a paradoxical competitive spirit and undermines solidarity. That is why Antonio defined modern society as “super competitive” and spoke of “a war among the poor,” and conversely, through the experience of his father, who was a blue-collar worker in the 1970's, he identified an opposite pattern:

“My father tells me that at that time there was the ability to hold a large mass of people together and that the unions led real struggles. While today we live in a competitive society that isolates and leaves people alone.”

From these sketches, we can see that in recent years the perception of work activity has changed profoundly, between forms of rejection, often on an individual basis, and a sense of impotence. This apparently contradictory situation, however, is based on a realistic approach that leads young people to concentrate more on the existential condition of the present and re-appropriation of themselves, often outside the dimension of “dependence” imposed on the single by the forms of contemporary accumulation/valuation.

## Some Preliminary Conclusions

In the previous sections we discussed how within the context of bio-cognitive capitalism, the two factors of valorization that appear to be among the most relevant are: the network value and the value of social reproduction. These two factors do not exhaust the problem of valorisation in contemporary capitalism. In fact, it is necessary to add to them also sources of value creation that refer to the traditional labor activity, increasingly permeated with knowledge. The labor value, in fact, is far from having disappeared. But, following the enlargement of the accumulation base, waged and hetero-direct labor is no longer the only way of extracting surplus value. We are thus faced with a heterogeneity of the processes of subsumption and exploitation of human life. The greater the hybridization between labor time and non-labor time, between human work performance and the machine element, between production and reproduction, the greater the complexity.

The two productive factors *par excellence* of capitalism, labor and capital, decompose and tend to mix between the tangible (machines, buildings, transport) and the intangible component (brand, learning, R&D), as far as capital is concerned, and between certified (and therefore remunerated) working time and uncertified (and therefore unpaid) productive life time, as far as labor is concerned. Networking and social relations, on the one hand, within the production organization by the social media and big data industries, and reproduction and care, on the other, within the modalities dictated by the biogenetic industry, health, prevention and welfare, social creativity and time management, thus become paradigms of a new accumulation regime.

The ambivalence of the current transformations leads to the need to redefine the salient factors that underlie both social cooperation as a source of accumulation and the forms in which such cooperation is captured and exploited by the new architecture of capitalist command. The concept of anthropomorphic capital, developed in paragraph 3, is paradigmatic from this point of view. It refers to the concept of human capital, developed by the Chicago school in the 1980's, but at the same time it irreversibly distances itself from it. If the idea, within the neoliberal thought of the Chicago school, was to show that between capital and labor there is no longer a conflicting dialectic but rather a synergy of growth of individual power, able to develop a universal entrepreneurial capacity, the version adopted here by the seminal studies of Cesarano shows us how the becoming human of capital is actually a new way of exploitation and expropriation of the sphere of life no longer enclosed in the increasingly narrow sphere of traditional wage work.

To better understand this aspect, it is also necessary to take up and remodel the philosopher's concept of subsumption (rather than strictly economic) developed by Marx in the 1844 Manuscripts. If the economic declination of this concept, the most purely economic terms is then developed by Marx in the two modes of formal subsumption (manufacturing system) and real subsumption (factory system), today this dichotomy tends to mix more and more, to the point of creating a subsumption process of a new nature, which is not limited to the length of the working day (formal subsumption), nor to the technological intensification of the themes of production (real subsumption).

Bio-cognitive capitalism is characterized by the simultaneous presence of formal subsumption and real subsumption at the same time. Formal subsumption, implicit in bio-cognitive capitalism, has to do with the redefinition of the relationship between productive work and non-productive work, making productive what was unproductive in the Fordist paradigm. The real subsumption has to do with the relationship between living and dead labor, as a consequence of the passage from repetitive mechanical technologies to linguistic and relational ones. Static technologies, at the base of productivity growth and work performance intensity (dimensional economies of scale), are transformed into dynamic technologies capable of exploiting learning and network economies, and simultaneously combining manual and relational activities. In recent years, the organization of work is increasingly reliant on the use of algorithms, able to directly organize a work activity, apparently characterized by a high degree of autonomy. The separation between execution and production of services is also becoming more difficult to analyse. They become inseparable within the production chain. As far as material production is concerned, the introduction of new computerized production systems requires professional skills and knowledge that make the relationship between man and machine increasingly inseparable, to the point that now living work can dominate the dead work of the machine, but within a new form of work organization and social governance. On the service production side (financialisation, research and development, communication, branding, marketing, personal services), we are witnessing a predominance of downstream valorisation, accompanied by a growing role of new forms of automation (based on algorithms).

In bio-cognitive capitalism, the real and the formal subsumption are thus two sides of the same coin and feed on each other. Together they create a new form of subsumption, which we can define as a vital subsumption, with reference not only to the sphere of knowledge and training, but also to the sphere of human relations, in the broadest sense.

The discussion on this subject is only just beginning.

## Data Availability Statement

Publicly available datasets were analyzed in this study. This data can be found here: H2020-ICT-2015, ICT10—Collective Awareness Platforms for Sustainability and Social Innovation (CAPSSI), Grant Agreement No. 687922: Title: PieNews-Commonfare.

## Ethics Statement

The studies involving human participants were reviewed and approved by H2020-ICT-2015, ICT10—Collective Awareness Platforms for Sustainability and Social Innovation (CAPSSI), Grant Agreement No. 687922: Title: PieNews-Commonfare. The patients/participants provided their written informed consent to participate in this study.

## Author Contributions

AF is professor of economics in the Department of Economics and Management at University of Pavia. He teaches also Theory of Firm at University of Bologna. He is member of Effimera Network, founder member of Bin-Italy (Basic Income Network, Italy. His latest book is: Cognitive Capitalism, Welfare, Labour, Routledge, London, 2019 (with A. Giuliani, S, Lucarelli, C. Vercellone). CM is a journalist, essayist, and independent researcher. She deals with issues related to gender and the processes of transformation of labour. She collaborates with various newspapers and websites. She is a founding member of the Bin-Italia association (Basic Income Network Italia) and of Effimera network. Her most relevant publications are The Feminilization of Labour in Cognitive Capitalism Feminist Review, vol. 87, 2007, 40–59, 2007; Per amore o per forza. Femminilizzazione del lavoro e biopolitiche del corpo, Ombre Corte, Verona 2010 and Lo sciopero delle donne. Lavoro, trasformazioni del capitale, lotte Manifestolibri, Rome, forthcoming 2019.

## Conflict of Interest

The authors declare that the research was conducted in the absence of any commercial or financial relationships that could be construed as a potential conflict of interest.

## Footnotes

^1^On the definition of *common* to singular, Michael Hardt, Antonio Negri, *Commonwealth*, Harward, Harward University Press, 2010, e Carlo Vercellone, Francesco Brancaccio, Alfonso Giuliani, Pierluigi Vattimo, *Il Comune come modo di produzione, Per una critica dell'economia politica dei beni comuni*, Ombre Corte, Verona, 2017, in particolar ch. 2. “We consider the common also and more significantly those results of social production that are necessary for social interaction and further production, such as knowledges, languages, codes, information, affect, and so forth,” M. Hardt, A. Negri, Commonwealth, Harward University Press, Cambidge, Mass., 2009, Usa, p. viii.

^2^Bio-cognitive capitalism (Fumagalli, 2019a) is preferable to more neutral locutions such as knowledge economy or knowledge-based economy to capture the inseparable hybridization between the development of productive forces and the development of social relations of production. It is thus intended to focus attention on the dialectical relationship between the two terms that make it up: - the term capitalism designates the permanence, even in their metamorphosis, of the fundamental variables of the capitalist system (the leading role of profit in the distribution of social work, wage labor, or rather, the forms of heterodirection of labor with respect to which surplus is extracted); - the bio-cognitive attribute highlights, instead, the new nature of work, the sources of valorization and the structure of property on which the process of accumulation is based and the contradictions that are generated there. In particular, bio-cognitive capitalism pays attention to the life directly put to value, not only through the intermediation of work, but also through the concept of “free labor.” As Terranova wrote in 2004 (p. 94): “Free labor is a desire of labor immanent to late capitalism and late capitalism is the field which both sustains free labor and exhausts it. It exhausts it by determining the means through which that labor can sustain itself: from the bum-out syndromes of internet start-ups to under-compensation and exploitation in the cultural economy at large.” We can say that free labor is an expression of the *common* when it is source of self-valorization and not of exploitation.

^3^For a more detailed analysis, see Jappe (2015), p. 113.

^4^This chapter refers to some analysis contained in Fumagalli et al. (2018).

^5^Data-mining is defined as “the set of techniques and methodologies that have as their object the extraction of a knowledge or a knowledge from large amounts of data (through automatic or semi-automatic methods) and the scientific, industrial or operational use of this knowledge”: https://en.wikipedia.org/wiki/Data_mining

^6^This network value should be distinguished from the scientific definition: according to Metcalfe's law, the “value” of each telecommunications network is directly proportional to the square of the number of nodes or users connected to the system.

^7^http://www.garzantilinguistica.it/ricerca/?q=cognitariato

^8^Pasquinelli (2015) writes: “The general intellect is therefore not only “crystallized” in the machines but spread through the entire “factory society” of the metropolis. Therefore, logically, if industrial knowledge designed and operated machines, even collective knowledge outside the factory must be somehow machinic. Here we have to look carefully at the manifestations of the general intellect through the metropolis to understand when we find it “dead” or “alive,” already “fixed” or potentially autonomous. For example, at what level today are the much celebrated Free Software and the so-called free culture complicit in the new forms of accumulation of digital capitalism? And at what level, do the ideology of creativity and Creative Cities simply prepare the ground for real estate speculation and new forms of metropolitan rents?”

^9^In this regard, the concept of Bio-ipermedia, coined by Giorgio Griziotti, is very interesting: “Bioipermedia can be broadly defined as the field in which the body in its entirety connects to network devices so intimate as to enter into a symbiosis in which changes and mutual simulations occur” (Griziotti, 2016, p. 120).

^10^This paragraph deals with some issues analysed in Morini (2019a,b).

^11^The fundamental point is in the concept of “labour power” introduced by Marx, according to which the worker does not sell his “work” to the capitalist, but rather himself as working capacity, for a certain number of hours a day. Marx writes: “By labour power or work capacity we mean the set of physical and intellectual attitudes that exist in the body, that is, in the living personality of a man, and that he sets in motion every time he produces values of use of any kind” (Marx, 2013, p. 200). For more details, see Ciccarelli (2018).

^12^Christian Marazzi speaks of “the emergence of an “*anthropogenetic model*.” According to him, the production of knowledge by means of knowledge is in fact a model of “*production of human being by means of human being*,” in which the possibilities of endogenous and cumulative growth have to do mainly with the development of the educational sector (investment in human capital), of the sectors of health (demographic evolution, biotechnologies, and of culture (innovation, communication, creativity). “In other words, the growth factors are ascribed directly to human activity…, that is, to the production of *life for*ms and, therefore, create of added value, which defines the human activity” (Marazzi, 2005).

^13^The interviews which are here partially reproduced are part of a broader research report (PIE News, 2017). These interviews are not the same of the original ones but modified for the Italian edition, but without changing the contents.

^14^www.Pienews.eu, www.commonfare.net

^15^H2020-ICT-2015, ICT10 — Collective Awareness Platforms for Sustainability and Social Innovation (CAPSSI), Grant Agreement No. 687922: Title: “PieNews-Commonfare” As Chiara Bassetti, one of the coordinators of the project for the University of Trento and leader of the research, describes: “The involvement and the active and repeated participation of people, groups and communities are therefore fundamental elements, both for the success and for the conduct of the research and co-planning project considered. Hence the centrality of the three “pilot” studies conducted in Croatia, Italy and the Netherlands thanks to and through the mediation of three partner organizations of the consortium. Each national study involves different categories of people and focuses on some cities. In particular, Museu de Crise (MdC) operates in the Netherlands, Amsterdam, Rotterdam and The Hague, and works with freelancers, welfare recipients and migrants; the association Basic Income Network - Italy (BIN) is active in Milan and Rome and focuses on precarious workers and unemployed young people; Finally, the Centar za MirovneStudije (CMS, Centre for Peace Studies) works in Croatia, especially in Zagreb but also in Rijeka, Split and Osijek, with the so-called NEETs, as well as in the archipelago, with the so-called island movement. Since October 2016, multi-sited research and co-design activities have been conducted in the three considered countries, through interviews, focus groups and design workshops. To date, more than 250 people have been involved and more than 50 collective meetings between focus groups and workshops have been held in the different local communities. Most of these communities have also been contacted on several occasions by researchers, researchers, designers, developers and developers from the other partner organizations of the consortium, which includes the University of Trento (Italy), project coordinator, Abertay University (Scotland), Madeira Interactive Technologies Institute (Portugal), Bruno Kessler Foundation (Italy) and Dyne.org, a “forge of free software” based in Amsterdam (Netherlands). A pilot case study for the commoncoin was conducted in Milan, with the self-managed group MACAO - New Centre for Arts, Culture and Research. The approach adopted is rooted in the tradition of participatory design, with which it shares the attention to practices endogenous to the context considered, the qualitative methods of ethnographic imprint, as well as the idea of an open and “endless” design process, based on the sharing of information and knowledge and on collective action, in a word, co-design (Bassetti, 2018, p. 22). The field research activity produced 252 in-depth individual interviews, 20 focus groups were organized, in order to investigate emerging social needs.

^16^Mangiarotti, in Corriere della Sera, July 16th, Mangiarotti (2009) “Malena, in her bedroom packed with books, nods: ≪I fight for what I like to do. So far so good. Maybe my parents and my old professor of literature do not feel at ease because they have always foreseen a “promising” future for me (what a bad word). Besides, our society does not accept those who seek a different way of living, far from a one-thousand-euro job as the one my sister got despite she is graduated and holds a PhD degree≫. ≪There was a chance at least – Daniele added – they stole it from me. My brother did everything to please this society and ended up without a job≫. Enrico B., 26 years old, does not work nor does he study but he has a girlfriend and a young son to look after: ≪My job? For months my job has been looking for a job. Now I take what comes≫. And who does look after the baby? ≪My mother and my father. For now we live with them, then we will see what happens≫.

^17^In this context, the authors use the term “*lightness*” to mean a condition characterized by reduced anxiety and a greater predisposition to face things as they are.
